# Dialysis Catheters and Their Common Complications: An Update

**DOI:** 10.1100/tsw.2009.145

**Published:** 2009-11-18

**Authors:** Satyaki Banerjee

**Affiliations:** Division of Nephrology, Louisiana State University Health Sciences Center, Shreveport, LA, USA

**Keywords:** hemodialysis, access, catheter, infection

## Abstract

Tunneled dialysis catheters (TDCs) are associated with the highest rate of complications, morbidity, and mortality when compared to arteriovenous fistulas or grafts, and this relates to higher costs in their management. Over time, catheters are prone to higher rates of infection, thrombosis, and central venous stenosis, and, thereby, catheter dysfunction. Lower blood flow rates are a consequence of the dysfuncion. Despite efforts to reduce incident and prevalent rates of catheter use for dialysis by the National Kidney Foundation and Fistula First Initiative, they remain a common modality of hemodialysis. The management of common TDC-related complications is discussed, in addition to ways to reduce and prevent morbidity associated with their use.

With the end-stage renal disease (ESRD) population increasing in the U.S., there has been an increasing trend towards maintenance hemodialysis as the modality of renal replacement. The preferred vascular access for hemodialysis is the arteriovenous fistula (AVF), with its high blood flow rate, and lowest infection and clotting problems. The arteriovenous graft (AVG) is the next preferred access, with blood flow rate comparable to the AVF. The catheters are rightfully the least preferred modality and, in an ideal setting, no patient should have a catheter as access. Despite the perils associated with dialysis catheters, their use has increased to almost 70% incident dialysis initiation with catheters[1].

The reason for that has been partly due to the increased emphasis on fistula creation by the "fistula first" initiative, leading to a decline in the number of new grafts being created, and more fistulas being immature and not usable for successful dialysis. Tunneled dialysis catheters (TDCs) are associated with the highest rate of complications, morbidity, and mortality when compared to AVFs or AVGs, and this relates to higher costs in their management[1]. Over time, catheters are prone to higher rates of infection, thrombosis, and central venous stenosis. Lower blood flow rates are a consequence of the thrombosis and stenosis. This, in turn, leads to lower Kt/Vs, poor clearance, and morbidity[2]. It has also been shown that catheters are an independent (of infection) inflammatory stressor and lead to increased morbidity[3].

These complications add to the burden of morbidity associated with ESRD itself. Catheters are usually a bridge access to a more permanent access like AVF, and sometimes the only access for some patients, and have to be used for months, if not years, for dialysis. The goal should be to provide safe, efficient, cost-effective, and sustained catheter function, while preserving future central access for AVF or

AVG creation. Herein, these potential complications associated with catheter use and how to best manage them are discussed.

## CAN WE GET BY WITHOUT A CATHETER?

This is a question we need to ask ourselves before planning any dialysis catheter placement. The best way to prevent complications later is to not insert a dialysis catheter in the first place. Alternate approaches, such as peritoneal dialysis or initiation with AVG followed by primary or secondary AVF creation, can be tried to avoid catheters. The patient, of course, has to be a candidate for the above alternatives and dialysis should not be emergently required in these scenarios.

## IN PREPARATION FOR CATHETER PLACEMENT

Once it is established that the patient needs a tunneled catheter for dialysis, the most crucial step is to prepare for the procedure in order to prevent complications during or after the procedure. Prior to surgical drape and scrub, an attempt should be made to look at the anatomy of the vessels in the neck with ultrasound probe. Stenosis or thrombosis can be detected even prior to an unsuccessful attempt[4]. During the procedure, this also helps in guiding the operator as to which side of the neck to go for and where to stick, thereby decreasing procedure time and complications. Attention should be paid to a full surgical scrub and drape to minimize infection rates.

The dialysis catheter that is used for long-term dialysis is universally tunneled under the skin for a segment of few inches and is called a TDC. The internal jugular vein is the preferred vein for cannulation, followed by the external jugular and femoral vein[1]. The subclavian vein should be absolutely avoided because of its higher rates of stenosis (60–70%)[5].

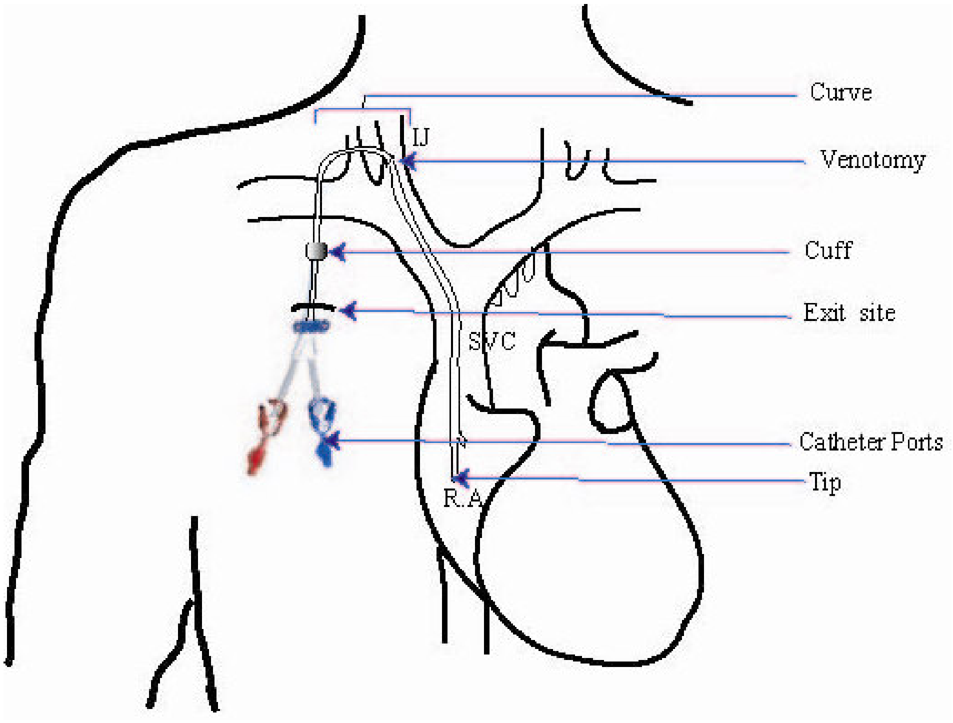

**FIGURE 1**. Appropriate tunnel catheter position and anatomy.

## INFECTION

Catheters are aptly referred to as a bacterial highway[6], and a biofilm formation around the catheter is often the breeding ground of bacteria and fungi, which can easily spread to the bloodstream.

Infectious complications are the most serious with regards to patient morbidity and mortality. The incidence could be from 3.8 to5.5 episodes per 1000 days[7]. Infection can be localized to just the exit site or can spread deeper into the tunnel (tunnelitis), or cause catheter-related bacteremia (CRB) systemically to trigger more serious consequences, such as sepsis, endocarditis, joint sepsis, vertebral abscess, discitis, or even mortality[8]. Symptoms can vary accordingly from localized pus discharge at exit site, redness, and catheter extrusion to fever, chills, and hemodynamic instability. It is dependent on the length of time the patient has the catheter and other factors, such as patient hygiene and precautions taken by the dialysis nurses during dialysis and the physicians during placement. There are different kinds of catheter-related infections and their management varies as per the severity[9].

- Exit site infection without discharge or any symptoms involves only the exit site and not the track. This can be treated with catheter exchange alone from the same exit site and track.
- Exit site infection with discharge or local symptoms should be treated with catheter exchange with creation of a new tunnel, but the same venotomy site can be used. A course of antibiotics should follow depending on cultures and sensitivities of pus discharge.
- Tunnel infection (tunnelitis) should be treated with removal of the old catheter and insertion of a new catheter at a new site altogether (new venotomy and exit site); otherwise, chances of bacteremia always exist. Antibiotics should again follow.
- CRB is defined as signs of catheter infection and bacteremia with two sets of positive blood cultures (peripheral and through catheter)[8]. The treatment should be prompt removal of the catheter along with a course of antibiotics, which can be broad spectrum at first, followed by specific antibiotics based on culture and sensitivity.

Use of an infected catheter, even for emergent dialysis, should be discouraged, as it would lead to seeding of bacteria in distant foci[9]. Ideally, the patient should be without a catheter for dialysis for 1–2 days prior to placement of a new catheter, while receiving antibiotics for 3 weeks. However, in the dialysis patient, the issue is complicated by the fact that the patient must continue to receive dialysis treatments[8]. Removal of the catheter creates a requirement for the use of temporary catheters and the risk of their associated complications. It mandates multiple procedures, a period of hospitalization, and increased costs. Additionally, removal of the catheter may be associated with a loss of the central venous entry site, a disastrous occurrence for a catheter-dependent patient[8]. Studies[10,11] have demonstrated that catheter exchange over a guide wire salvaged 80–88% of sites without apparent ill effects. It has been found that a negative culture result is not required before catheter exchange[10] and there is no advantage in delaying replacement of the catheter by several days[11].

## PROPHYLAXIS OF INFECTION

Given the huge burden on ESRD patient morbidity, mortality, and health care costs, it only makes sense to take some simple steps when trying to prevent infection. Asepsis and proper scrubbing and handling of catheters in dialysis units should be done with attention to prevent contamination, as well as the wearing of masks and gloves by both nurses and patients. Proper cleansing of the catheter hubs should be done prior to catheter hookup. The exit site should also be cleansed with 70% alchohol and chlorhexidine[8]. Use of local mupirocin ointment should be encouraged at the exit site until the site heals after placement of the catheter. Manipulation of the catheter during or after dialysis should be kept to a minimum[8]. After dialysis, the catheter and exit site should be cleansed and dressed appropriately. Every effort should be made to transition to an AVF or AVG for permanent access, whenever possible, and the catheter should be taken out at the earliest possible time.

## CATHETER DYSFUNCTION

This is a very common problem associated with the chronic use of catheters. The National Kidney Foundation (NKF/DOQI) published guidelines stating that dysfunction is defined as failure to attain a sufficient extracorporeal blood flow of 300 ml/min, with a prepump arterial pressure more negative than 250 mmHg[12]. Thrombosis and sheath formation, whether intra- or periluminal, has been reported as the primary reason for catheter dysfunction, with 17–33% requiring untimely removal of the catheter[13]. This, consequently, is attributed to access loss in 30–40% of patients[12].

Dysfunction leads to improper clearance on dialysis, leading to lower Kt/V and higher morbidity. For every 0.1 drop in Kt/V, there is an 11% rise in hospitalization and increase in health care costs[1].

Once recognized, the catheter dysfunction should be managed with a trial of thrombolytic therapy[14].

A milligram of tPA diluted with saline can be instilled in each port of the catheter and 15–20 min given to dissolve the intracatheter clot. Periodically, more saline should be pushed to advance the thrombolytic forward towards the tip. After a total of about 30 min, the two ports should be aspirated. If successful, the catheter should be flushed properly at this stage. If thrombolysis is unsuccessful, the patient should be referred for angiography to check for fibroepithelial sheath formation. The sheath is a reaction to the foreign body (catheter in the vein) with a biomembrane made of cells, fibrin, proteoglycans, and cell debris. If a sheath is present, the catheter should be removed, percutaneous balloon angioplasty performed to break the sheath and dilate the venous channel, and then a new catheter (catheter exchange) should be replaced over wire[2].

Central venous stenosis is also a major complication associated with catheters. The longer a patient has a catheter, the greater are the chances of development of central stenosis. The neck, face, and ipsilateral side of the chest are usually swollen and collateral veins can be seen on the surface of the chest and neck. Stenosis requires removal of the catheter and percutaneous balloon angioplasty of the involved segment of the vein[15]. Usually, if the catheter is replaced in the same vein, this problem can recur.

## CATHETER LOCK SOLUTIONS

Recent literature has shown promise regarding the concept of prevention of catheter infection and clotting using the lock solution with 30% citrate and gentamicin[16]. Thirty percent citrate serves as an effective anticoagulant and a moderately strong antibacterial. The combination of the two can be used in the two ports of the catheters after dialysis. Further prospective randomized studies exploring their efficacy are under way[16].

## CATHETER EXTRUSION

At times, patients have an “exposed cuff” or even extreme outcomes, such as “catheter falling out”. Infection of the exit should be investigated and addressed if present. If not infected, the catheter can be replaced over wire without creation of new tunnel or track[17]. The new catheter can be wider in diameter than the cuff, thereby securing it better to subcutaneous tissue, or additional subcutaneous sutures can be applied to hold the catheter at the exit site. Over time, new adhesions develop with the cuff and prevent the catheter from extruding[17].

## WHICH TYPE OF CATHETER TO USE?

There are numerous companies and different types of TDCs. There is no perfect catheter. Every operator has a preference for a specific kind with time. Of all the parameters of a catheter, the two most important are length and diameter. The bigger the diameter of the catheter, the less would be the resistance to blood flow. On the other hand, the longer the catheter, the more would be the resistance to blood flow. Therefore, an optimal catheter should be as wide and as short as feasible for the patient.

## SUMMARY

It is well known and recognized that catheters are not optimum for long-term dialysis because of their high morbidity and mortality. Greater emphasis should be placed on pre-ESRD education to create AVFs in patient for incipient dialysis and thereby minimize tunneled catheter use[18]. The complications of infections and catheter dysfunction with thrombosis and sheath formation are the most common causes of patient morbidity, and relate to extra health care expenditure. Efforts should be made to prevent these complications and to address them at the earliest. It is important to understand that the complications of infection and dysfunction are a vicious cycle and are often interrelated. Thrombosis and fibrin sheaths both enhance central CRB by providing an interface for adherence and colonization. If the catheter is used as a bridge until the patient gets a fistula, it should be ensured that the patient gets the AVF or AVG placed as soon as possible and when usable, the catheter should be taken out[18]. Complications such as thrombosis, infection, stenosis, and catheter dysfunction arise with the use of TDCs and should be addressed at the earliest in order to decrease patient morbidity and lower health care costs[9,14].
